# The Radical Cure of Aveolar Abscess

**Published:** 1883-03

**Authors:** S. J. Hutchinson


### The Radical Cure of Alveolar Abscess.

BY S. J. HUTCHINSON, M.R.C.S., ETC.

[Read before the Odontological Society of Great Britain.]

Mr. President and Gentlemen: I cannot enter upon the subject of my paper, without a few words of explanation as to how so familiar a topic has been chosen. At an early meeting of last Session, March or April, it was decided to have an evening set apart for discussion, without a formal paper, and I was asked by the officers just to start the above subject. However, the supply of papers was so full that no opportunity for such a discussion offered itself, and I willingly held myself ready for any time when a blank should occur.

As I have already said, by the activity of our officers no such blank occurred, and at the last meeting of the Society it was decided that at the November meeting I should read a short paper on "The Radical Cure of Alveolar Abscess."

Constantly before my mind has been the fact that the paper was to be read to a society of practitioners well skilled in their art, and not to mere tyros, and that, therefore, copious extracts on the pathology of alveolar abscess would be out of place. I have, therefore, confined myself to acknowledging most of the sources from which the following notes are taken, and giving also a few cases, illustrative of a plan which seems to give the keynote to a greater success in its treatment.

I will quote from Taft's "Operative Dentistry," 3rd Edit., just published, wherein he admits: "In regard to the treatment of alveolar abscess much remains to be learned. With the attainments thus far made in this direction no aspiring dentist will rest satisfied, though in the hands of a few it has made great progress within a very recent period." This book has only been published a few months, and is the most recent authority, yet the following plan is not alluded to, although in the November number of the *Cosmos*, 1876, Dr. Farrar gave some of its details.

Last session two-thirds of the papers were about the complications of the nerve-pulp, alive and dead, and since April there has been time to collect cases, with which I trust we may be favored. Our President in February, as also Mr. Barrett, in December, 1875, said that "he hoped a categorical list of cases would settle the matter finally." I, unaided, cannot hope to do this, but wish Mr. Barrett's idea may be realized. Mr. Coleman, Mr. Ashley Barrett, Dr. Waker, and Mr. Spence Bate all alluded to the cure of alveolar abscess in their papers, and spoke of creosote, arsenic, and carbolic acid as the agents; and Mr. Underwood, Dr. Field and Mr. Stocken each described their methods, using creosote, salicylic, and carbolic acids.

In looking over the old Transactions, in 1861 I find our President wrote a paper on fang-filling, before the discovery of carbolic acid; and alcohol was the purifying agent, with creosote. In May, 1865, Mr. Woodhouse introduced the subject of carbolic acid as a dressing for exposed nerves, and predicted the usefulness it has since attained; and in November, 1865, Mr. Thomas Rogers's paper was on fang-filling, and he used creosote and iodine as a dressing, saying he often found obscesses disappear, he did not know how or why. Mr. James Bate, in February, 1876, had a paper on the carbolic treatment.

The most obvious and the most successful cure of alveolar abscess is the extraction of the offending tooth, but such a course is radical indeed, and we must, if possible, inaugurate a "conservative-radical" treatment, but the cases must be chosen judiciously for the teeth to be "preserved usefully,"

The upper incisors and bicuspids and the lower temporary molars are those which have been most successfully treated by me, and I will later give the cases, and the time they were under treatment.

The term "preserved usefully" is used advisedly, because a tooth kept in the head on which the patient cannot eat, is worse than to extract it, for the obvious reason that it cripples the whole side it belongs to. The lesser evil of a tooth senitive to heat and cold is more to be condoned, as this system is wont to disappear; but, personally, I must confess I have worn out my patients' endurance, and my own, in the endeavor to render hopeless teeth helpful, and I am almost losing heart at the uphill work it is.

Although the means I shall shortly detail have proved helpful in many cases, yet the failures are numerous, and I think a broad line of distinction may be drawn, dividing two classes,—1st. Where a fistulous opening or chronic "gum-boil" is present. 2nd. Where this does not exist, but there are symptoms of periostitis and latent alveolar abscess.

These two classes each contain all the successes and failures—the successful cases where a "gum-boil" exists, the unsuccessful ones where the disease has not progressed so far. And to the former division I would chiefly devote my paper, seeing that my experience hitherto has been favorable; and on the other division I would crave the members present to freely debate, as there is much to learn on this point.

This part of the paper will not contain any original matter, as it must be admitted the plan adopted has been published before, and, indeed, referred to in this room, but the manner of applying the dressing is a little different, and, I believe, more advantageous.

To take a typical case—say a central incisor, of which the pulp has come to grief from the application of an osteo-stopping, the the most common cause—and it will be found that the cervical edge of the stopping has wasted away, and the pulp is dead and decomposed, with a small chronic abscess over the apex of the fang. Here we have a most favorable specimen to treat, provided excavation of the caries and the stopping leaves the tooth a good color.

Having removed every particle of pulp-remains from the fang as far as is possible without going through the apex of the fang, a light dressing of aconite and chloroform should be applied up the root. This dressing of aconite and chloroform is well worthy of note as an application when the pulp cavaties have been cleaned out, but all periostitis not subsided, for it acts then like a charm in removing the tenderness of a tooth in its socket.

A chief point to observe in order to gain success, is to be more than careful of the perfect cleanliness of the barbs and broaches used in clearing out the fang. This seems a small matter, but too much care cannot be used, when one remembers that Professor Lister, at King's College, said, "that a needle-point dipped in a very weak solution of decomposed blood and water, and then that needle-point only allowed to touch the edge of a pure vessel of blood, would develop in it crowds of bacteria!

On the next day but one the treatment may commence (after putting on the rubber-dam) by the application of pure crystals of carbolic acids, Calvert's No. 1, into the pulp cavity, and here is the point I desire to be noted—that no water or glycerine should be added as a solvent of the acid, the heat of the tooth in a few seconds being sufficient to melt the crystals, and to insure the absence of germs by any diluent. The crystals are most easily applied on a fragment of amadou to which they cling, and do not drop about the mouth.

A piece of wool should be wrapped round a flexible broach, so as to form a sort of piston-rod the size of the pulp cavity, and being saturated with carbolic acid it will not absorb that already in the tooth, but if the piston be worked up and down will force the acid well up the tooth, and possibly through the apex of the fang, so as to appear on the surface of the gum through the fistulous opening—this I have been able to do on a few occasions in the most successful cases. When this has been thoroughly performed the cavity may be syringed out with warm water, and the same process repeated. The tooth may now be considered ready for filling, and during the preparations the whole cavity should contain pure carbolic acid on wool in excess, and it is well to keep the rubber-dam on all the time, as it keeps the acid in due bounds. A fresh minute piece of cotton is taken soaked in the pure acid, having also ready several broaches with clean wool on them to dry out the chamber, then with a fine instrument pass up the carbolized wool to the apex of the fang, and next an equally small piece of wool immersed in a very liquid mixture of osteo, as thin as cream, and the wool loosely wrapped, so as to be imbued with the fluid; this next goes up the fang as quickly as possible, the object being to seal up an atmosphere which shall be anti-septic. The remainder of the fang can now be filled with fluid osteo, with filaments of wool in it to ensure its being well carried up the root.

The cavity of decay, when the osteo is hard, may be filled with gutta-percha stopping, as being the most easily applied and easily removed in case of bad consequences.

It is maintained that by having all particles of pulp, by any chance not removed, thoroughly saturated in carbolic acid, and then sealed up hermetically in that condition, there is less likelihood of a bad result from any access of atmospheric air, the germs of which should cause putrescence. There is thus inaugurated what has been justly termed "conservative antiseptic dentistry," and the conditions here exist for the perfect hindrance of decay, and I may be pardoned if I wander from the point to remark, that the great desideratum (yet, alas! undiscovered) is a stopping of the nature of osteo which shall be applied similarly, but which shall not wear away under mastication, for it would fulfil every requirement of the patient, and most certainly prevent the spread of caries, besides removing all fear of electro-chemical action from the mixture of gold and amalgam fillings.

And I must further crave your pardon, when I say that if a reliable stopping can be put in a tooth in half-an-hour, it is not to the patient's interest to spend two over it.

Papers have been read before this Society on the various phases of alveolar abscess, but the text books do not give this particular method of treatment.

Mr. Coleman has tried the removal of the tooth, excision of the abscess, and replantation. I hope we shall hear of his success. Mr. Spence Bate recommends glycerine applied like carbolic acid, and Dr. Farrar, in the paper in *Cosmos*, November 1876, uses a special syringe adapted by himself, which ejects creosote or aromatic sulphuric acid down the fangs or into the fistula. Taft proposes the somewhat heroic treatment of trephining the bone over the apex of the diseased fang and excising the abscess

Others, again, practice a method of establishing a more perfect sinus by means of a seton or plug, so as to allow the abscess to heal out from the bottom.

So far the treatment has depended on a condition of chronic abscess opening on the gum surface; but before this stage is reached, it is necessary to have some means at command to save the tooth, and instead of using carbolic acid, I recommend creosote and morphia mixed into a strong paste, for the heat of the tooth liquefies this sufficiently to let it go well up the fangs; and a wonderfully soothing effect it seems to have—the creosote is antiseptic and the morphia anodyne; by this treatment a tender tooth, raised in its socket, may, by a few weeks' careful dressing with creosote and morphia, be made ready to stop with every chance of success.

Mr. Coleman, at one meeting, spoke of the use of arsenic as a permanent dressing at the bottom of large and old cavities in refilled teeth; it would be interesting to know whether he still finds this application successful.

With regard to cases I find most amenable to treatment, I can quote several of upper incisors and bicuspids; but except in the case of children, I do not find the molars particularly subservient to treatment.

Case I.—Miss. M. Left upper lateral dark in color; pulp dead; chronic abscess; gold stopping. Removed stopping; cleaned out fang; applied pure carbolic on wool without attempting to force it through the apex of fang on first day. In two days changed dressing and put on rubber-dam, which, by the way, should always be applied; filled the pulp-cavity with crystals of pure carbolic acid; saturated also a plug of wool which would just fill cavity like a piston-rod; pumped it up and down, and was able to see the gum blanch at the fistulous opening, showing that the acid had worked right through. I now put a fresh piece of wool soaked in carbolic, about the size of millet seed, at the extreme apex of fang, to prevent air being forced through the fang; then, mixing some osteo very thin and involving in it shreds of wool. I proceeded to fill the fang perfectly air-tight and antiseptic, nearly to the pulp-chamber; this I filled with Hill's stopping, and at the end of a week removed this and put in gold, the tooth having much improved in color and appearance, and the fistula quite disappeared.

Case II.—Mrs. T. Right upper first bicuspid, with chronic sinus. Here, on cleaning out remains of pulp, it was followed by inflammation of periosteum and swelling of cheek and eyelids; but patient was determined to persevere, so after a longer period of dressing (about a fortnight) with carbolic acid, the tooth became ready for filling exactly as described, only the gutta-percha has not yet been replaced by a harder material.

Case III.—Master H. R. Acute abscess at roots of first temporary molars; age five years; parents and myself very anxious to keep them their due time.

The abscess pointed over the roots and discharged freely, and by clearing out the remains of the pulp and dressing with carbolic acid, which in this case came out through fistula, I was in a fortnight able to fill them both, and six months afterwards they were quite healthy and firm in their sockets.

I think I have here given sufficient evidence of the success of this method, though my case-book contains others, and in my hands, at least, the pure carbolic acid treatment with perfectly air-tight and antiseptic stopping, seems best adapted for the radical cure of alveolar abscess.

